# Bacterial Extracellular Vesicles as Regulators of the Gut Microbiome–Circadian Axis in Inflammatory and Metabolic Diseases

**DOI:** 10.4014/jmb.2607.07032

**Published:** 2026-08-26

**Authors:** Hyejin Choi, Sangnam Oh, Younghoon Kim

**Affiliations:** 1 Department of Agricultural Biotechnology and Research Institute of Agriculture and Life Science, Seoul National University, Seoul 08826, Republic of Korea; 2 Department of Functional Food and Biotechnology, Jeonju University, Jeonju 55069, Republic of Korea

**Keywords:** Bacteria-derived extracellular vesicle (BEVs), Gut microbiome, Circadian rhythm, Inflammatory diseases, Metabolic diseases

## Abstract

Bacteria-derived extracellular vesicles (BEVs) have emerged as key mediators of intercellular and interkingdom communication. These nanoscales, lipid bilayer–enclosed particles are secreted by both Gram-positive and Gram-negative bacteria and carry diverse bioactive cargoes, including proteins, lipids, nucleic acids, and metabolites. Through the delivery of these molecules, BEVs regulate host physiology by modulating immune responses, epithelial barrier integrity, and gut microbial community composition. Accumulating evidence indicates that BEVs derived from probiotics or commensal bacteria promote gut homeostasis, whereas vesicles released by pathogenic bacteria exacerbate dysbiosis, inflammation, and disease progression. The gut microbiome plays a central role in maintaining immune and metabolic homeostasis, and its disruption contributes to the development of inflammatory and metabolic diseases, including inflammatory bowel disease, atopic dermatitis, and metabolic dysfunction–associated steatohepatitis. In parallel, circadian rhythms orchestrate host metabolism, immunity, and microbial interactions, and emerging evidence highlights a bidirectional relationship between the gut microbiome and the circadian clock. Disruption of this microbiome–circadian axis further exacerbates inflammation and metabolic dysfunction. This review integrates current knowledge of the structural and functional characteristics of BEVs, their roles in regulating the gut microbiome, and their emerging involvement in circadian biology. We further discuss how BEVs may mediate communication within the gut microbiome–circadian axis and contribute to the pathogenesis or resolution of inflammatory and metabolic diseases. Understanding the BEV–gut microbiome–circadian axis provides new mechanistic insights into host–microbe communication and highlights BEVs as promising postbiotic candidates for restoring immune–metabolic homeostasis and developing next-generation microbiome-targeted therapeutics.

## Introduction

Bacteria-derived extracellular vesicles (BEVs) have emerged as key mediators of host–microbe communication and are increasingly recognized as an additional signaling layer linking the gut microbiome with host physiology. These nanoscale, lipid bilayer-enclosed particles are secreted by both commensal and pathogenic bacteria and carry diverse bioactive cargoes, including proteins, lipids, nucleic acids, and metabolites, that mediate intercellular and interkingdom communication [1, 2]. Through the coordinated delivery of these molecules, BEVs regulate immune responses, epithelial barrier integrity, and gut microbial ecology, thereby influencing both local and systemic host physiology [3]. While BEVs derived from probiotics and commensal bacteria promote gut homeostasis by alleviating dysbiosis and suppressing inflammation, vesicles released by pathogenic bacteria may exacerbate disease progression [4, 5]. Beyond their established role in host–microbiome communication, accumulating evidence suggests that BEVs may also contribute to temporal regulation by linking microbial activity with the host circadian system [6, 7]. Recent studies further indicate that BEV-mediated signaling participates in the gut microbiome–circadian axis by transmitting microbial information that influences host metabolism, immune function, and epithelial homeostasis [7, 8]. Disruption of this temporal communication, as occurs during circadian misalignment or gut dysbiosis, can exacerbate inflammatory and metabolic disorders, including inflammatory bowel disease (IBD), atopic dermatitis (AD), and metabolic dysfunction–associated steatohepatitis [7, 9]. Unlike soluble microbial metabolites, which generally transmit individual biochemical signals, BEVs simultaneously deliver proteins, lipids, nucleic acids, and metabolites within a single vesicle [1]. This coordinated mode of cargo delivery distinguishes BEVs from soluble microbial mediators and may enable the simultaneous regulation of multiple host signaling pathways, providing an additional regulatory layer within host–microbiome communication and a conceptual basis for microbiome-targeted therapeutics and next-generation postbiotic strategies.

Circadian rhythms are intrinsic timekeeping systems that synchronize physiological and behavioral processes with the 24-h light–dark cycle. The central pacemaker located in the suprachiasmatic nucleus (SCN) coordinates peripheral clocks distributed throughout nearly all tissues, including the gastrointestinal tract, through hormonal, neural, and metabolic signals [10]. In the intestine, circadian oscillations regulate epithelial renewal, nutrient absorption, mucus secretion, and immune surveillance, thereby preserving mucosal integrity and metabolic homeostasis [11]. Disruption of these rhythms through genetic defects, irregular feeding patterns, or environmental disturbances results in chrono-disruption, compromising gut barrier function and promoting inflammation and metabolic dysfunction [9]. Accumulating evidence demonstrates that the gut microbiota exhibits robust diurnal oscillations in composition, gene expression, and metabolite production, functioning both as a downstream effector and an upstream regulator of host circadian rhythms [12]. Microbial metabolites, including short-chain fatty acids, bile acids, and tryptophan derivatives, serve as peripheral zeitgebers that entrain host clocks and synchronize intestinal and systemic metabolism [13]. Conversely, host circadian transcription factors such as *BMAL1* and REV-ERBα modulate microbial rhythmicity by regulating nutrient availability and immune tone [7]. These bidirectional interactions constitute the gut microbiome–circadian axis, which maintains temporal homeostasis between microbial activity and host physiology. Beyond soluble microbial metabolites, BEVs have emerged as an additional signaling platform capable of protecting and delivering diverse molecular cargoes, raising the possibility that vesicle-mediated signaling contributes to the transmission of rhythmic microbial information to host tissues [12]. Despite growing evidence supporting the role of BEVs in host–microbe communication, whether they directly participate in circadian regulation remains largely unexplored [14]. This knowledge gap limits our understanding of how rhythmic microbial signals are integrated into host physiology and disease progression. Addressing this question is essential for establishing the mechanistic basis through which microbial temporal information is transmitted to the host and may facilitate the development of microbiome-based chronotherapeutic strategies [15].

This review proposes a conceptual framework in which BEVs function as signaling mediators linking the gut microbiome with host circadian regulation in inflammatory and metabolic diseases. We summarize current knowledge of BEV biology, their roles in host–microbiome communication, and the emerging evidence supporting their involvement in circadian regulation. By integrating advances in BEV biology, gut microbiome research, and circadian physiology, we discuss how vesicle-mediated signaling represents an additional regulatory layer within the gut microbiome–circadian axis. We further examine how this emerging concept provides mechanistic insight into inflammatory and metabolic diseases and discuss its implications for chronobiology-informed microbiome therapeutics and next-generation postbiotic strategies. Finally, we propose that BEVs represent a previously underappreciated signaling platform that integrates microbial rhythmicity with host circadian regulation, thereby providing a new conceptual framework for understanding host–microbe interactions and developing precision microbiome-targeted interventions.

## Molecular Composition of Bacteria-Derived Extracellular Vesicles

### Biogenesis of BEVs

**Fig. 1:**
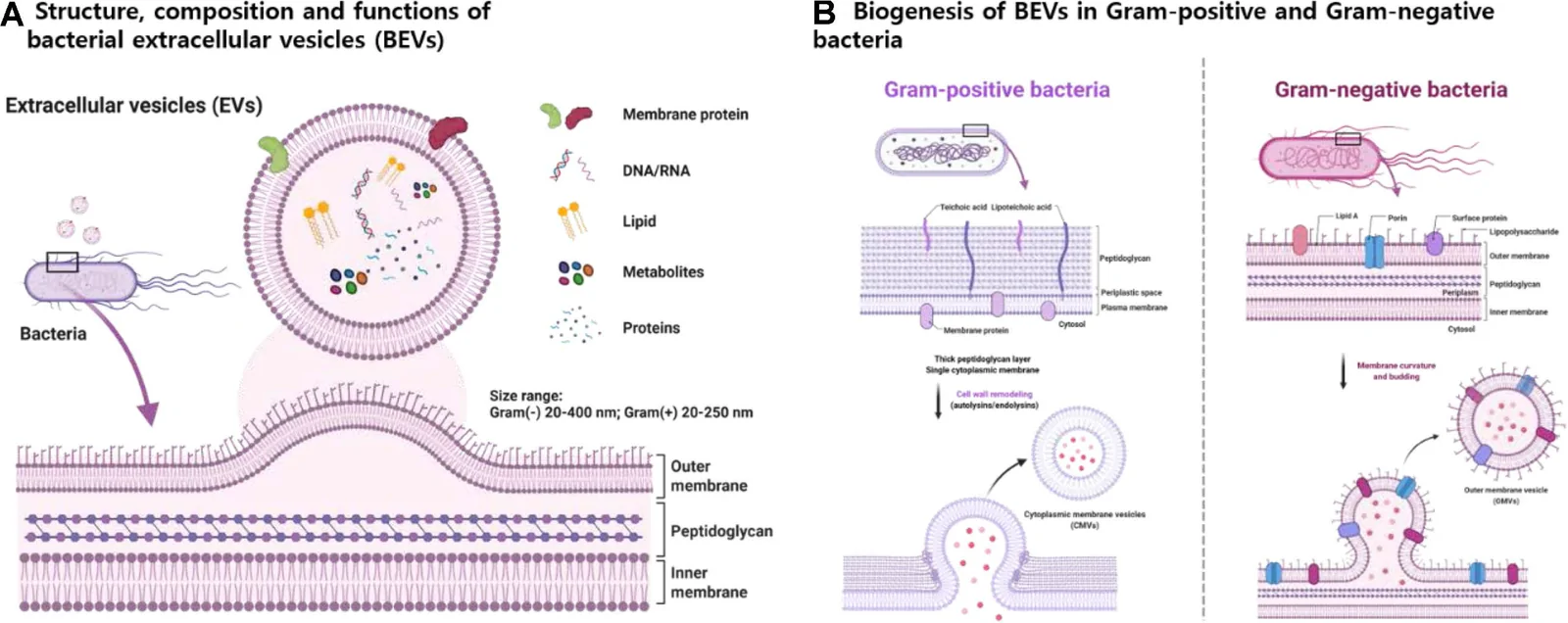
Structural organization, molecular composition, and biogenesis of bacterial extracellular vesicles (BEVs). (**A**) Structural organization and molecular composition of bacterial extracellular vesicles (BEVs). BEVs are nanoscale lipid bilayer-enclosed vesicles released by bacteria that encapsulate diverse bioactive cargoes, including proteins, lipids, nucleic acids (DNA and RNA), metabolites, and membrane-associated molecules. The lipid bilayer protects these cargoes from extracellular degradation and enables their coordinated delivery to recipient bacterial and host cells, thereby facilitating intercellular and interkingdom communication. The schematic illustrates the general architecture of BEVs and their major molecular components. (**B**) Biogenesis of BEVs in Gram-positive and Gram-negative bacteria. Gram-positive bacteria generate cytoplasmic membrane vesicles (CMVs) through localized remodeling of the thick peptidoglycan layer, allowing membrane protrusion and vesicle release. Gram-negative bacteria produce OMVs through membrane curvature, budding, and blebbing of the outer membrane. These distinct mechanisms of vesicle biogenesis reflect differences in bacterial cell envelope architecture and contribute to the characteristic molecular composition and biological functions of BEVs.

Extracellular vesicles (EVs) have emerged as a highly conserved mechanism of intercellular communication across all domains of life, including eukaryotes, bacteria, and archaea [16, 17]. These nanoscale, lipid bilayer-enclosed particles protect diverse bioactive cargoes, including proteins, lipids, nucleic acids, and metabolites, from extracellular degradation while enabling efficient transfer of biological information between neighboring and distant cells (Fig. 1A) [1, 18]. In eukaryotic systems, EVs are generally classified into exosomes (30–150 nm), microvesicles (100–1000 nm), and apoptotic bodies (500–2000 nm) according to their biogenesis, molecular composition, and biological functions [19]. Although these vesicle populations differ in their cellular origin and mechanisms of formation, all contribute to immune regulation, tissue homeostasis, and adaptation to environmental stress. Among these vesicle populations, BEVs have attracted increasing attention as key mediators of host–microbiome communication. Produced by both Gram-negative and Gram-positive bacteria, BEVs typically range from 20 to 400 nm in diameter and are generated through coordinated membrane remodeling rather than passive membrane fragmentation or bacterial lysis (Fig. 1B) [20]. This controlled mode of biogenesis enables bacteria to selectively package biologically active cargoes into membrane vesicles that subsequently interact with neighboring microorganisms as well as host cells. These unique structural and biogenetic properties distinguish BEVs from soluble microbial mediators and establish them as highly organized signaling platforms for microbial communication and host–microbe interactions.

The mechanisms underlying BEV biogenesis differ depending on bacterial cell envelope architecture. In Gram-negative bacteria, BEVs are commonly referred to as outer membrane vesicles (OMVs) and are generated through outward bulging and subsequent pinching-off of the outer membrane [21]. Because OMVs originate from the outer membrane, they encapsulate outer membrane components together with periplasmic contents, including lipopolysaccharides (LPS), phospholipids, outer membrane proteins, enzymes, and periplasmic proteins [22]. OMVs biogenesis is now recognized as a dynamic and tightly regulated process rather than constitutive membrane shedding. Environmental stimuli, including envelope stress, oxidative stress, nutrient availability, antibiotic exposure, and quorum sensing, have all been shown to influence vesicle production and cargo composition, allowing Gram-negative bacteria to dynamically adapt extracellular communication to changing environmental conditions.

In contrast, Gram-positive bacteria lack an outer membrane and possess a thick peptidoglycan layer that was initially believed to prevent extracellular vesicle release [23]. Subsequent studies demonstrated, however, that Gram-positive bacteria actively produce membrane vesicles (MVs) through localized remodeling of the peptidoglycan layer mediated by autolysins and other cell wall–modifying enzymes, thereby permitting protrusion of the cytoplasmic membrane and subsequent vesicle release [24]. These vesicles contain membrane-associated and cytoplasmic proteins, phospholipids, nucleic acids, and metabolites that reflect the physiological characteristics of the parental bacterium [25]. The discovery that both Gram-negative and Gram-positive bacteria actively release extracellular vesicles has substantially expanded our understanding of biological significance of BEVs [1]. Although the molecular composition of BEVs varies among bacterial species, accumulating evidence indicates that cargo incorporation is not simply a passive consequence of vesicle formation. Instead, proteins, lipids, nucleic acids, and metabolites appear to be selectively enriched in response to bacterial physiological status and environmental conditions. Such selective cargo loading enables bacteria to dynamically modify vesicle composition according to physiological demands, thereby optimizing communication with neighboring microorganisms and host tissues [26]. Therefore, these findings indicate that BEVs function as highly regulated extracellular signaling systems rather than random membrane fragments.

Beyond vesicle biogenesis, the biological significance of BEVs is ultimately determined by the molecular cargo delivered to recipient bacteria and host cells. BEVs participate in bacterial communication, horizontal gene transfer, immune modulation, epithelial barrier maintenance, and regulation of microbial community dynamics. These diverse biological activities are largely dictated by the molecular cargo packaged within individual vesicles, emphasizing that vesicle biogenesis and cargo selection are tightly coordinated processes that ultimately determine BEVs function [27]. Current evidence supports the concept that BEVs are highly organized extracellular signaling platforms rather than passive by-products of bacterial growth. Their biological functions depend not only on their bacterial origin but also on the molecular mechanisms governing vesicle biogenesis and selective cargo loading [28]. Accordingly, understanding the molecular composition of BEVs provides the conceptual basis for the following discussion of BEV cargo composition and its functional implications.

### Molecular Composition and Functional Cargo of BEVs

The biological functions of BEVs are largely determined by their molecular cargo, which is selectively incorporated during vesicle biogenesis. Encapsulation within a lipid bilayer protects diverse bioactive cargoes, including proteins, lipids, nucleic acids, and metabolites, from extracellular degradation while enabling the coordinated delivery of multiple signaling molecules to recipient bacteria and host cells [29-31]. As a result, the molecular composition of BEVs not only determines their structural properties but also underlies their diverse biological functions in microbial communication, host–microbe interactions, and immune regulation.

Lipids constitute the structural scaffold of BEVs and play essential roles in vesicle stability, membrane integrity, and cellular interactions [32]. The lipid composition of BEVs is primarily derived from the bacterial membrane and varies according to bacterial cell envelope architecture. In Gram-negative bacteria, BEVs contain outer membrane-derived phospholipids, LPS, and membrane-associated proteins, whereas Gram-positive BEVs are enriched in cytoplasmic membrane phospholipids and lipoteichoic acids [33]. Beyond providing structural support, these membrane lipids influence membrane curvature, vesicle stability, cellular uptake, and immunogenicity. Together, these properties determine how BEVs interact with recipient bacteria and host cells and ultimately influence their biological activity [34].

Proteins represent one of the most functionally diverse components of BEV cargo. Proteomic analysis have identified a broad spectrum of proteins, including enzymes, transport proteins, adhesins, toxins, stress-response proteins, and immunomodulatory molecules. Notably, several studies have demonstrated that specific proteins are selectively enriched in BEVs relative to their parental bacterial cells, indicating that protein incorporation is an actively regulated process rather than a passive consequence of vesicle formation. This selective protein loading enables bacteria to dynamically modify vesicle function in response to environmental conditions and contributes to microbial communication, host–pathogen interactions, immune modulation, and environmental adaptation [35, 36].

Nucleic acids, including genomic DNA, plasmid DNA, messenger RNA (mRNA), and small regulatory RNAs, are increasingly recognized as biologically active components of BEVs [26]. Delivery of these nucleic acids to recipient bacteria or host cells can alter gene expression, facilitate horizontal gene transfer, and promote the dissemination of antibiotic resistance, virulence factors, and metabolic traits [37]. Beyond mediating genetic exchange between bacteria, vesicle-associated RNAs may also participate in interkingdom communication. Although the functional significance of many BEV-associated nucleic acids remains to be fully elucidated, accumulating evidence suggests that they provide an additional layer of regulatory communication beyond conventional soluble microbial mediators.

EVs are a broad class of membrane-enclosed extracellular particles released by cells. In this review, BEVs specifically refer to EVs derived from bacteria. Thus, BEVs represent a bacteria-derived subset of the broader EV category. In addition to macromolecules, EVs encapsulate a wide variety of low-molecular-weight metabolites, including amino acid derivatives, quorum-sensing molecules, secondary metabolites, and other small signaling compounds [38]. These metabolites contribute to bacterial adaptation, microbial communication, and environmental sensing, where they act synergistically with proteins and nucleic acids to regulate biological responses. Unlike freely diffusible metabolites, vesicle-associated metabolites are protected within a membrane-bound structure, enabling more efficient delivery to recipient cells and potentially enhancing their biological activity.

Collectively, the molecular composition of BEVs defines their biological functions and distinguishes them from soluble microbial mediators [39]. By coordinating the delivery of proteins, lipids, nucleic acids, and metabolites, BEVs function as highly organized extracellular signaling platforms that integrate microbial communication with host physiological regulation. As discussed in the following sections, these molecular characteristics enable BEVs to influence epithelial barrier integrity, immune homeostasis, microbial ecology, and ultimately the gut microbiome–host communication network [40]. Understanding the molecular composition and selective cargo loading of BEVs therefore provides a critical foundation for elucidating their roles in host–microbiome communication and for developing standardized BEV isolation methods, functional characterization approaches, and next-generation microbiome-targeted therapeutic strategies.

## Isolation and Characterization of Bacteria-Derived EVs

### Isolation Methods

Reliable isolation of BEVs is a prerequisite for accurately defining their molecular composition, biological functions, and therapeutic potential. Because BEVs are released into complex bacterial culture media containing intact bacterial cells, protein aggregates, membrane fragments, extracellular polymers, and other non-vesicular components, isolation strategies must efficiently recover vesicles while preserving their structural integrity and minimizing contamination. Consequently, the choice of isolation method directly influences vesicle yield, purity, molecular composition, and the interpretation of downstream functional and multi-omics analyses. To address these challenges, a variety of isolation approaches have been developed, including differential centrifugation, density gradient centrifugation, ultrafiltration, size-exclusion chromatography (SEC), and polyethylene glycol (PEG)-based precipitation, each with distinct advantages and limitations (Table 1) [25, 41].

Differential centrifugation remains the most widely used and fundamental approach for BEV isolation [42]. This method employs sequential centrifugation steps to remove intact bacterial cells and large debris, followed by ultracentrifugation at 100,000–150,000 × *g* to pellet vesicles [17]. Although differential centrifugation is cost-effective and widely accessible, protein aggregates, membrane fragments, and other extracellular particles are frequently co-isolated, thereby reducing sample purity. Consequently, differential ultracentrifugation alone is often insufficient for obtaining highly purified BEV preparations. To improve purity, density gradient centrifugation using sucrose or iodixanol is commonly employed as an additional purification step [43]. By separating vesicles according to buoyant density, this approach produces highly purified preparations suitable for ultrastructural, proteomic, and other omics analyses, although it is relatively time-consuming and requires specialized equipment.

Ultrafiltration and SEC provide alternative or complementary approaches that separate vesicles according to particle size [44]. Ultrafiltration enables rapid concentration of BEVs but may induce vesicle deformation because of shear stress. In contrast, SEC provides gentle separation with high reproducibility while minimizing contamination from soluble proteins. However, it frequently generates dilute fractions that require additional concentration before downstream analysis [45].

PEG-based precipitation has become a practical alternative because of its simplicity, scalability, and high recovery efficiency [46]. By reducing vesicle solubility through volume-exclusion effects, PEG enables rapid enrichment of BEVs without ultracentrifugation. However, PEG precipitation may also co-isolate soluble proteins, extracellular polysaccharides, and other non-vesicular particles, potentially influencing downstream proteomic and functional analysis [47]. Accordingly, PEG precipitation is frequently combined with washing procedures or complementary purification methods, such as SEC, to improve sample purity while maintaining vesicle recovery.

No single isolation method is universally optimal for all bacterial species or experimental objectives. Instead, isolation strategies should be selected according to the intended downstream application while balancing vesicle yield, purity, structural integrity, reproducibility, and scalability. Standardized isolation protocols will be essential for enabling meaningful comparisons across studies and for supporting the increasing application of BEVs in multi-omics research, biomarker discovery, and therapeutic development. Equally important, rigorous isolation procedures provide the foundation for accurate molecular characterization, which is critical for understanding the biological functions and translational potential of BEVs.

### Molecular Characterization of BEVs

Following isolation, comprehensive molecular characterization is essential for verifying the identity, purity, structural integrity, and biological properties of BEVs. Because no single analytical technique can fully characterize these heterogeneous vesicle populations, complementary approaches are typically combined to evaluate vesicle morphology, particle size, concentration, molecular composition, and biological activity. Such multidimensional characterization is particularly important for distinguishing authentic BEVs from protein aggregates, membrane fragments, or other extracellular contaminants. Transmission electron microscopy (TEM) remains one of the most widely used techniques for visualizing BEVs morphology [48]. TEM enables direct observation of vesicle size and membrane architecture, thereby confirming the presence of intact lipid bilayer-enclosed particles. Cryogenic electron microscopy (cryo-EM) has further advanced structural characterization by preserving vesicles in their native hydrated state, minimizing artifacts introduced during sample preparation and providing higher-resolution structural information [49].

Nanoparticle tracking analysis (NTA) is routinely employed to determine particle size distribution and concentration [50]. Tracking the Brownian motion of individual particles enables quantitative estimation of vesicle size distribution and concentration. Dynamic light scattering (DLS) can also be used to estimate particle size; however, its accuracy decreases in heterogeneous samples because larger particles disproportionately influence scattering intensity. While imaging and biophysical techniques define vesicle morphology and physical properties, molecular characterization provides insight into BEVs composition and biological function. Recent advances in proteomics, lipidomics, metabolomics, and transcriptomics have demonstrated that BEVs selectively package diverse proteins, lipids, nucleic acids, and metabolites that reflect the physiological state of the parental bacterium [51, 52]. High-resolution mass spectrometry enables comprehensive profiling of vesicular proteins, lipids, and metabolites, whereas next-generation sequencing facilitates detailed analysis of vesicle-associated DNA and RNA. Together, these multi-omics approaches provide mechanistic insights into selective cargo loading and the molecular basis of host–microbiome communication [53]. Beyond structural and molecular characterization, functional validation has become an essential component of BEVs research [54]. Cellular uptake assays, epithelial barrier models, immune cell stimulation assays, organoid systems, and animal disease models are widely employed to evaluate the biological activities of isolated BEVs. Integrating structural, molecular, and functional analyses not only improves interpretation of vesicle-mediated biological effects but also enhances reproducibility and facilitates meaningful comparisons across independent studies.

Collectively, comprehensive BEV characterization requires the integration of complementary imaging, biophysical, molecular, and functional approaches rather than reliance on any single analytical technique. Such integrated characterization provides the foundation for reproducible BEV research, enables mechanistic investigation of host–microbiome communication, and supports the development of BEV-based diagnostics, postbiotics, and microbiome-targeted therapeutics.

### Current Application and Challenges of BEVs

Despite substantial methodological advances, several challenges continue to limit the reproducibility, standardization, and clinical translation of BEVs research [55]. At present, BEVs are increasingly being explored as biomarkers, postbiotic candidates, therapeutic delivery vehicles, and mediators of host–microbiome communication. However, the rapid expansion of these applications has also highlighted several methodological limitations that must be addressed before their biological and therapeutic potential can be fully realized.

First, considerable variability exists among isolation protocols, making direct comparisons across challenging studies. Different isolation strategies frequently generate vesicle preparations that differ in yield, purity, molecular composition, and functional activity, leading to inconsistent biological outcomes. Such methodological variability is particularly problematic when comparing studies investigating immune regulation, microbiome modulation, or therapeutic efficacy. A second major challenge is the absence of universally accepted BEV-specific markers. Unlike mammalian extracellular vesicles, for which several consensus markers have been established, BEVs exhibit substantial heterogeneity depending on bacterial species, growth conditions, and cell envelope architecture. Thus, comprehensive characterization using complementary imaging, molecular, and functional approaches remains essential for validating BEVs preparations.

The increasing application of multi-omics technologies has further emphasized the importance of standardized sample preparation and quality control. Proteomic, lipidomic, metabolomic, and transcriptomic analyses are highly sensitive to sample purity, and contamination from bacterial debris, extracellular proteins, or non-vesicular particles may substantially influence molecular profiling. Accordingly, standardized workflows encompassing isolation, characterization, quality assessment, and data reporting will be essential for improving reproducibility and facilitating meaningful comparisons across laboratories. Looking forward, integrating standardized isolation procedures with advanced multi-omics analyses and rigorous functional validation will provide a more comprehensive understanding of BEV biology. Such efforts will strengthen mechanistic studies while accelerating the development of BEV-based diagnostics, postbiotic therapeutics, and engineered vesicle platforms [56]. Ultimately, these methodological advances will establish the experimental foundation required to elucidate how BEVs regulate host physiology through the gut microbiome and how vesicle-mediated signaling contributes to the gut microbiome–circadian axis in inflammatory and metabolic diseases.

## Functional Roles of BEVs in Host–Microbiome Communication

### BEVs as Mediators of Host–microbiome Communication

BEVs have emerged as highly organized signaling platforms that mediate communication between the gut microbiota and host tissues. Unlike intact bacteria, BEVs readily traverse biological barriers while protecting their diverse molecular cargo within a lipid bilayer. This unique mode of cargo delivery enables proteins, lipids, nucleic acids, and metabolites to be transferred to recipient cells in a coordinated manner, thereby extending the functional influence of the gut microbiota beyond the intestinal lumen [57]. Consequently, BEVs have become recognized as key mediators of intercellular and interkingdom communication that integrate microbial activity with host physiological regulation.

Within microbial communities, BEVs facilitate bacterial communication by transporting quorum-sensing molecules, enzymes, antimicrobial compounds, and genetic material. These cargoes regulate biofilm formation, nutrient acquisition, microbial competition, and horizontal gene transfer, thereby promoting microbial community stability and enabling rapid adaptation to changing environmental conditions [58-60]. Beyond their roles within microbial communities, BEVs actively interact with epithelial and immune cells. BEVs derived from commensal and probiotic bacteria generally preserve epithelial barrier integrity, regulate innate and adaptive immune responses, and maintain intestinal homeostasis (Table 2) [61, 62]. In contrast, pathogenic BEVs frequently carry toxins, adhesins, lipopolysaccharides, and other virulence-associated molecules that promote epithelial injury, inflammatory signaling, and tissue damage [63]. These contrasting biological effects emphasize that the functional properties of BEVs are determined largely by their bacterial origin and selective cargo composition.

A defining characteristic of BEVs is their remarkable stability under physiological conditions. Encapsulation within a lipid bilayer protects vesicular cargo from enzymatic degradation while facilitating transport through biological fluids, thereby enabling communication between the gut microbiota and distant organs [64]. This unique delivery capability has attracted increasing interest in the development of BEV-based therapeutics, including postbiotic interventions, vaccine platforms, targeted drug delivery systems, and immunomodulatory agents [29, 65]. Collectively, current evidence supports the concept that BEVs function as highly organized extracellular signaling platforms rather than passive transport particles. By coordinating the delivery of diverse bioactive cargoes, BEVs regulate epithelial barrier integrity, immune homeostasis, and microbial ecology while providing an additional signaling layer within the gut microbiome–host communication network.

### Regulation of Gut Barrier Integrity Via BEVs

The intestinal epithelial barrier represents the primary interface between the gut microbiota and the host, maintaining selective permeability while preventing the translocation of pathogens and harmful microbial products. Increasing evidence indicates that BEVs play a central role in preserving intestinal barrier integrity by regulating epithelial proliferation, mucus production, tight junction organization, and epithelial repair [4]. BEVs derived from probiotic and commensal bacteria enhance epithelial barrier function through multiple complementary mechanisms. They promote the expression of tight junction proteins, including ZO-1, occludin, claudins, and mucin-associated proteins, thereby reducing intestinal permeability and preserving mucosal integrity [66]. In addition, BEVs stimulate epithelial renewal and intestinal stem cell activity, facilitating tissue regeneration following inflammatory injury [67]. Beyond their direct effects on epithelial cells, BEVs indirectly reinforce barrier function by modulating immune responses and reshaping the gut microbial community. Increased production of anti-inflammatory cytokines, suppression of excessive NF-κB activation, and enrichment of beneficial bacterial populations collectively attenuate chronic mucosal inflammation and promote epithelial homeostasis [68]. Through these coordinated actions, BEVs contribute to the restoration and maintenance of barrier integrity under pathological conditions, including inflammatory bowel disease and metabolic disorders. In contrast, BEVs released by pathogenic bacteria frequently compromise epithelial barrier integrity. These vesicles deliver toxins, proteases, and other virulence-associated molecules that disrupt tight junction proteins, increase epithelial permeability, and facilitate bacterial invasion [69]. The resulting barrier dysfunction promotes systemic inflammation by increasing the translocation of microbial components and inflammatory mediators into the circulation. Collectively, maintenance of epithelial barrier integrity represents one of the principal mechanisms through which BEVs regulate host physiology. By integrating epithelial function, immune signaling, and microbial ecology, BEVs establish a dynamic communication interface between the gut microbiota and the host.

### Immunomodulatory Functions via BEVs

Unlike intact bacteria, BEVs directly interact with host immune cells without requiring bacterial colonization or invasion, enabling rapid modulation of both innate and adaptive immune responses. Their nanoscale size and membrane-bound structure facilitate access to epithelial and immune cells while protecting and delivering diverse bioactive cargo [70]. BEVs have emerged as important mediators that maintain immune homeostasis at the host–microbiome interface [71]. BEVs derived from commensal and probiotic bacteria generally exert immunoregulatory effects by promoting immune tolerance while limiting excessive inflammatory responses. Probiotic-derived BEVs stimulate the production of anti-inflammatory cytokines, including interleukin-10 (IL-10), while suppressing pro-inflammatory mediators such as tumor necrosis factor-α (TNF-α), interleukin-1β (IL-1β), and interleukin-6 (IL-6) through modulation of the NF-κB and mitogen-activated protein kinase (MAPK) signaling pathways [72, 73]. These coordinated responses promote resolution of intestinal inflammation and preserve mucosal immune homeostasis.

Beyond cytokine regulation, BEVs influence the activity of multiple immune cell populations. Macrophages, dendritic cells, neutrophils, and intestinal epithelial cells recognize BEV-associated microbial components through pattern-recognition receptors (PRRs), including Toll-like receptors (TLRs) and nucleotide-binding oligomerization domain (NOD)-like receptors, thereby initiating signaling cascades that coordinate innate immune responses [74]. Under physiological conditions, controlled activation of these pathways supports immune surveillance, tissue repair, and maintenance of intestinal homeostasis, whereas excessive activation contributes to chronic inflammation. BEVs also modulate adaptive immunity through vesicle-associated antigens that are internalized and processed by antigen-presenting cells, leading to T-cell activation and regulation of regulatory T-cell responses [75]. Recent studies further suggest that probiotic-derived BEVs preferentially promote immune tolerance by reinforcing regulatory immune networks rather than sustaining chronic inflammatory activation, highlighting their therapeutic potential as naturally derived immunomodulatory agents. In contrast, BEVs released from pathogenic bacteria frequently carry virulence-associated molecules, including lipopolysaccharides, toxins, proteases, and adhesins, which trigger excessive inflammatory responses and tissue injury [76]. These vesicles activate NF-κB signaling, inflammasome pathways, and pro-inflammatory cytokine production, thereby exacerbating epithelial damage and disease progression. Together, these findings demonstrate that the immunological consequences of BEV exposure are determined largely by bacterial origin and selective cargo composition.

Taken together, BEVs function as dynamic regulators of innate and adaptive immunity by integrating microbial signals with host immune responses. Through balanced regulation of inflammatory signaling, immune tolerance, and tissue homeostasis, BEVs establish an important communication network between the gut microbiota and the host immune system. This immunomodulatory capacity represents a central mechanism by which BEVs maintain host–microbiome homeostasis and influence both intestinal and systemic physiology.

### Regulation of Gut Microbial Ecology via BEVs

In addition to regulating host physiology, BEVs actively shape the composition, functional activity, and ecological stability of the gut microbiome [47, 77-79]. Through the transfer of proteins, nucleic acids, metabolites, and signaling molecules, BEVs mediate communication within microbial communities and coordinate diverse ecological stability [5]. Unlike direct bacterial interactions that require physical proximity, vesicle-mediated signaling enables the transfer of biological information across greater distances, thereby facilitating coordinated regulation of microbial behavior throughout the intestinal ecosystem. BEVs participate in multiple ecological processes that determine microbial community structure and function. Vesicle-associated enzymes and metabolites influence nutrient utilization and metabolic cooperation, whereas antimicrobial proteins and competitive signaling molecules suppress opportunistic pathogens and shape niche competition [80]. In addition, BEV-mediated horizontal gene transfer facilitates the dissemination of genetic traits, including antibiotic resistance genes and metabolic capabilities, thereby promoting bacterial adaptation to changing environmental conditions.

Increasing evidence indicates that BEVs derived from beneficial bacteria selectively promote microbial communities associated with intestinal health. Administration of probiotic-derived BEVs increases the abundance of beneficial taxa, including *Akkermansia*, *Lactobacillus*, and *Bacteroides*, while reducing the relative abundance of inflammatory or opportunistic microorganisms [4, 74, 81, 82]. These compositional changes are frequently accompanied by improvements in epithelial barrier integrity, immune homeostasis, and metabolic function, suggesting that modulation of the gut microbiome represents an important mechanism underlying the beneficial effects of BEVs. Beyond altering microbial composition, BEVs also regulate microbial function [83]. Vesicle-mediated signaling influences microbial metabolism, quorum sensing, biofilm dynamics, bile acid transformation, and the production of bioactive metabolites, including short-chain fatty acids and tryptophan-derived metabolites. Because these metabolites function as key signaling molecules within the gut–host axis, BEV-mediated regulation of microbial metabolism has the potential to influence host physiology beyond the intestine. Collectively, BEVs influence both the taxonomic composition and functional capacity of the gut microbiome, thereby maintaining ecosystem stability while facilitating host–microbiome communication. By coordinating microbial ecology with host physiological responses, BEVs provide an important mechanistic framework for understanding how microbial signals are integrated across the gut–host axis. This ecological perspective serves as a conceptual foundation for the following section, which explores how BEV-mediated host–microbiome communication may extend to circadian regulation.

### BEVs as Integrated Signaling Platforms

Unlike conventional microbiome-derived metabolites, which generally transmit individual biochemical signals, BEVs encapsulate multiple classes of bioactive molecules within a single membrane-bound structure, including proteins, lipids, nucleic acids, and metabolites. This unique molecular organization enables the coordinated delivery of diverse functional cargoes, allowing multiple biological pathways to be regulated simultaneously rather than independently [84]. Accordingly, BEVs are better viewed as integrated signaling platforms than simple extracellular transport vehicles. The membrane architecture of BEVs provides several biological advantages over freely diffusible microbial metabolites. Encapsulation protects vesicular cargoes from enzymatic degradation, enhances molecular stability within the gastrointestinal environment, and facilitates efficient uptake by epithelial cells, immune cells, and other recipient tissues [85]. Following internalization through endocytosis, membrane fusion, or receptor-mediated uptake, multiple cargo molecules can be delivered simultaneously into host cells, thereby activating interconnected signaling pathways that collectively regulate cellular physiology. This coordinated mode of signaling distinguishes BEVs from soluble postbiotic molecules that generally mediate more restricted biological effects.

The biological activity of BEVs depends not only on individual cargo molecules but also on their integrated molecular composition. Proteomic analysis have identified enzymes, adhesins, stress-response proteins, and immunomodulatory factors that regulate host immunity and epithelial function, whereas lipidomic studies demonstrate that membrane lipids contribute to vesicle stability, cellular uptake, and inflammatory signaling [86]. In parallel, BEV-associated DNA, mRNA, and small RNAs influence gene expression in recipient microorganisms and host cells, while encapsulated metabolites further modulate microbial metabolism and host physiological responses [51]. By integrating proteins, lipids, nucleic acids, and metabolites within a single vesicle, BEVs orchestrate epithelial barrier integrity, immune homeostasis, microbial ecology, and metabolic signaling as interconnected biological processes rather than independent events. This integrated mode of regulation provides a mechanistic explanation for the broad biological activities of BEVs and underscores their central role in host–microbiome communication.

Notably, recent studies indicate that many host pathways regulated by BEVs including immune responses, epithelial barrier function, microbial metabolism, and cellular energy homeostasis, are themselves under circadian regulation [87, 88]. Likewise, the gut microbiome exhibits pronounced diurnal oscillations in microbial composition and metabolite production that influence host physiology [89]. These observations raise the possibility that BEVs function not only as spatial mediators of host–microbiome communication but also as temporal signaling platforms capable of transmitting rhythmic microbial information. Although direct experimental evidence remains limited, this emerging concept provides a compelling mechanistic framework for investigating the proposed BEV–gut microbiome–circadian axis.

**Fig. 2:**
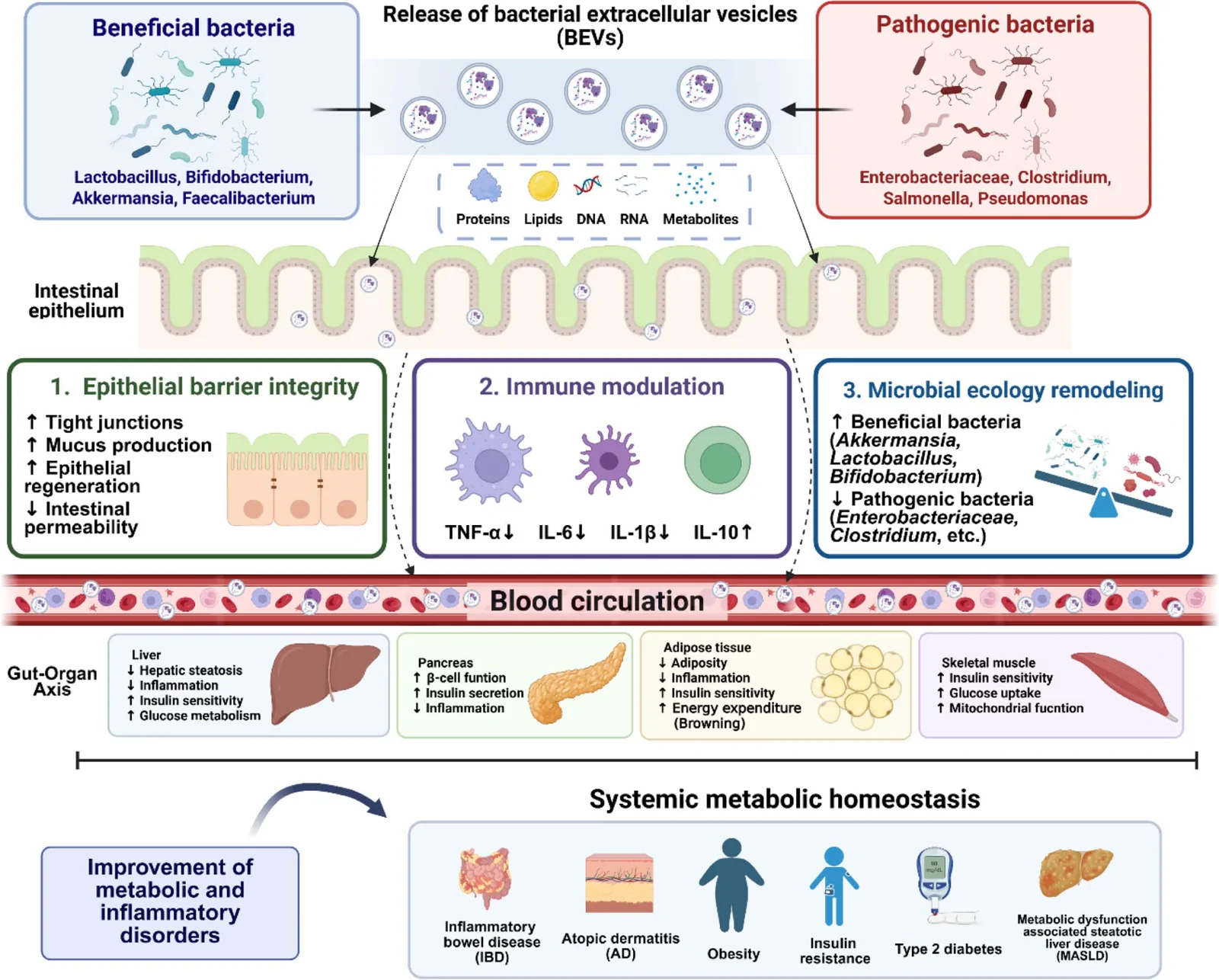
Bacterial extracellular vesicles coordinate host–microbiome communication along the gut–organ axis. Gut microbiota-derived bacterial extracellular vesicles (BEVs) are released by both commensal and pathogenic bacteria into the intestinal lumen. BEVs encapsulate diverse bioactive cargoes, including proteins, lipids, nucleic acids, and metabolites, within a lipid bilayer that enables coordinated delivery of multiple signaling molecules to recipient cells. Following interaction with the intestinal epithelium, BEVs regulate epithelial barrier integrity, immune homeostasis, and microbial ecology by promoting beneficial microbial communities while suppressing pathobionts [136]. A proportion of BEVs subsequently translocates across the intestinal barrier into the systemic circulation, where they communicate with peripheral organs, including the liver, pancreas, adipose tissue, skeletal muscle, and other metabolically active tissues. Through coordinated regulation of epithelial function, immune responses, microbial ecology, and systemic metabolism, BEVs contribute to host physiological homeostasis and may alleviate inflammatory and metabolic disorders, including inflammatory bowel disease (IBD), atopic dermatitis (AD), obesity, insulin resistance, type 2 diabetes mellitus (T2DM), and metabolic dysfunction-associated steatotic liver disease (MASLD).

Therefore, current evidence suggests that BEVs coordinate epithelial barrier integrity, immune responses, microbial ecology, and systemic metabolism through interkingdom signaling. As these biological processes are themselves under robust circadian regulation, BEVs may contribute not only to spatial communication along the gut–organ axis (Fig. 2) but also to the temporal coordination of host physiology [53]. The following section therefore examines the emerging evidence linking BEV-mediated host–microbiome communication to circadian regulation in health and disease.

## BEVs as Mediators of the Gut Microbiome–Circadian Axis

### The Gut Microbiome–circadian Axis

**Fig. 3:**
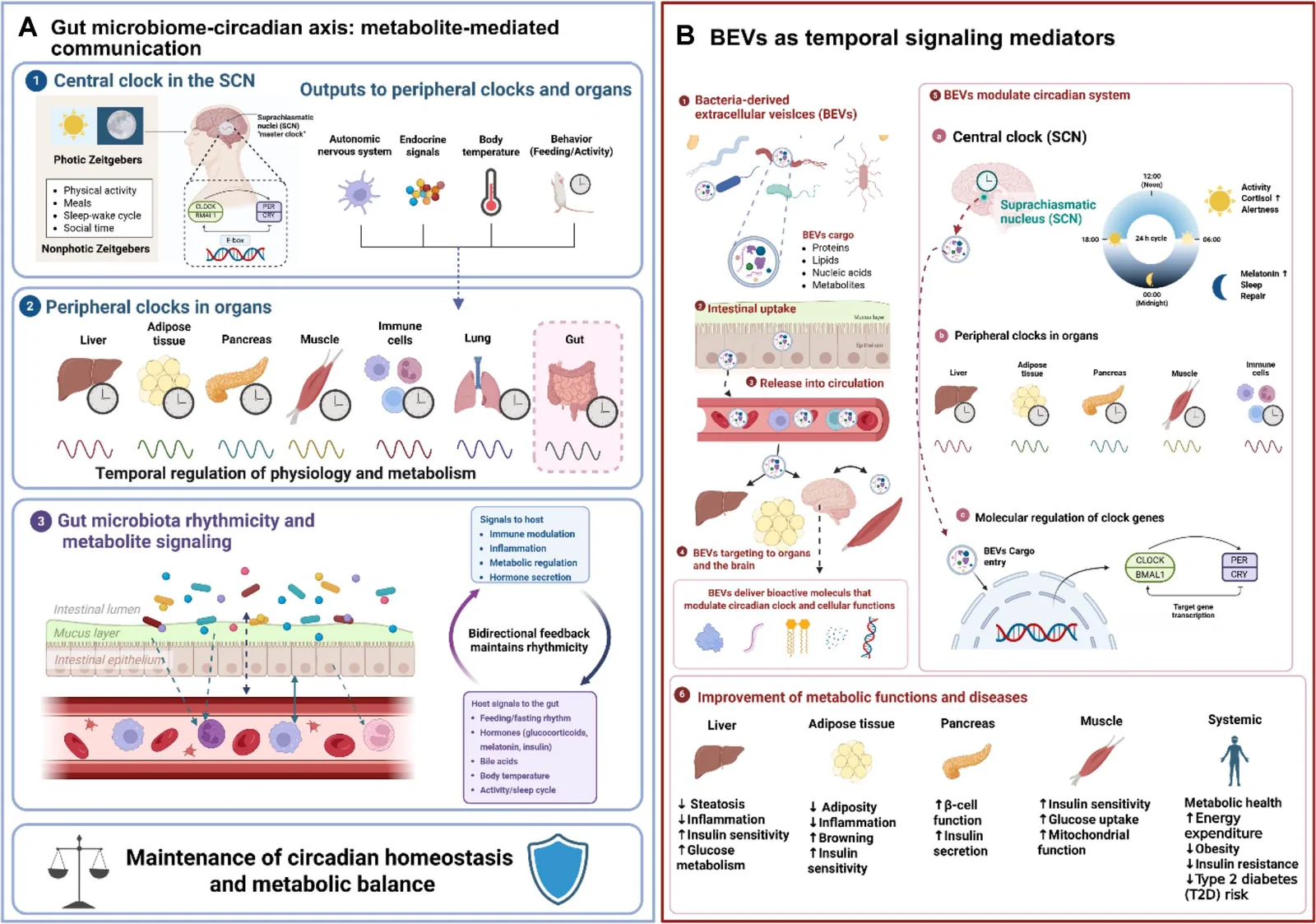
Bacterial extracellular vesicles integrate microbial rhythmicity with host circadian regulation. (**A**) Bidirectional communication between the gut microbiome and the host circadian system. The central circadian pacemaker located in the suprachiasmatic nucleus (SCN) synchronizes peripheral clocks through endocrine, neural, metabolic, and behavioral cues. Circadian rhythms regulate feeding behavior, immune function, hormone secretion, body temperature, and intestinal physiology, thereby shaping gut microbial composition and rhythmic microbial metabolism. In turn, microbiome-derived signaling molecules including short-chain fatty acids, bile acids, tryptophan metabolites, and other microbial metabolites, provide feedback to peripheral tissues and contribute to circadian and metabolic homeostasis. (**B**) Proposed role of BEVs as emerging temporal signaling mediators within the gut microbiome–circadian axis. Rhythmic microbial activity is hypothesized to influence BEV biogenesis, selective cargo loading, and vesicle-mediated signaling. Following release into the intestinal lumen, BEVs traverse the intestinal barrier, enter the systemic circulation, and communicate with peripheral organs and circadian regulatory networks. By coordinating the delivery of proteins, lipids, nucleic acids, and metabolites, BEVs are proposed to integrate microbial rhythmicity with host circadian signaling, thereby regulating epithelial barrier integrity, immune homeostasis, microbial ecology, and metabolic function. Although direct experimental evidence remains limited, this conceptual model highlights BEVs as integrated signaling platforms that may coordinate temporal host–microbiome communication and contribute to inflammatory and metabolic disease homeostasis.

The circadian system coordinates nearly all aspects of mammalian physiology through a hierarchical network consisting of the central clock located in the SCN of the hypothalamus and peripheral clocks distributed throughout multiple organs, including the gastrointestinal tract, liver, adipose tissue, skeletal muscle, and immune cells [90]. This circadian timing system synchronizes physiological and behavioral processes with the 24-h light–dark cycle through coordinated oscillations of core clock genes, including, including Brain and Muscle ARNT-Like 1 (*BMAL1*), Circadian Locomotor Output Cycles Kaput (*CLOCK*), Period (*PER1–3*), and Cryptochrome (*CRY1–2*) [8]. These molecular clocks regulate diverse biological processes, including epithelial renewal, nutrient absorption, immune surveillance, hormone secretion, and energy metabolism, thereby maintaining physiological homeostasis (Fig. 3) [91].

Circadian rhythms were traditionally considered to be governed primarily by host-derived mechanisms. However, accumulating evidence has fundamentally changed this view by demonstrating that the gut microbiome constitutes an integral component of the circadian system rather than merely a passive responder to host rhythmicity. The intestinal microbiota exhibits robust diurnal oscillations in microbial abundance, community composition, transcriptional activity, and metabolic function that are synchronized with host feeding behavior and the light–dark cycle. High-throughput sequencing and metatranscriptomic analyses have further revealed that numerous bacterial taxa undergo time-dependent fluctuations in abundance, while microbial metabolic pathways involved in carbohydrate fermentation, amino acid metabolism, and bile acid transformation also display pronounced circadian rhythmicity throughout the day [92].

Host circadian rhythms and the gut microbiota communicate through a bidirectional regulatory network. On one hand, host circadian clocks regulate feeding behavior, intestinal motility, mucus secretion, antimicrobial peptide production, epithelial turnover, and nutrient availability, thereby establishing rhythmic environmental conditions that shape microbial community structure and function [9, 93]. Genetic disruption of core clock genes such as *Bmal1* and *Clock*, alters microbial composition and diminishes microbial rhythmicity, demonstrating that host circadian regulation directly influences the intestinal microbial ecosystem. Conversely, the gut microbiota actively regulates host circadian physiology through the production of bioactive metabolites and signaling molecules. These microbiome-derived factors function as peripheral *zeitgebers* that synchronize intestinal and systemic clocks by modulating circadian gene expression in metabolic tissues [94]. Their influence extends beyond the intestine to distant organs, including the liver, adipose tissue, pancreas, and immune system, thereby coordinating whole-body metabolic homeostasis. Together, these findings establish the gut microbiome as an active component of the circadian network that both responds to and shapes host rhythmic physiology.

Disruption of this reciprocal interaction results in chrono-disruption, a condition characterized by impaired synchronization between host circadian rhythms and microbial oscillations. Environmental factors, including shift work, jet lag, irregular feeding schedules, sleep deprivation, and high-fat diets, disturb microbial rhythmicity, leading to reduced microbial diversity, altered metabolite production, intestinal barrier dysfunction, and chronic low-grade inflammation [92]. These alterations have been associated with IBD, obesity, metabolic dysfunction-associated steatotic liver disease (MASLD), type 2 diabetes mellitus (T2DM), cardiovascular diseases, and other chronic inflammatory disorders, underscoring the physiological importance of maintaining temporal coordination between the gut microbiota and the host circadian system [90, 95, 96].

Current evidence suggests that communication within the gut microbiome–circadian axis is mediated by diverse microbiome-derived signaling molecules rather than by microbial composition alone [89]. Among these, short-chain fatty acids (SCFAs), secondary bile acids, tryptophan metabolites, and other microbial metabolites have been extensively investigated as key mediators linking microbial metabolism with host circadian regulation. BEVs have emerged as another potential signaling platform capable of transporting multiple classes of bioactive cargo simultaneously. Unlike conventional soluble metabolites, BEVs package proteins, lipids, nucleic acids, and metabolites within a stable lipid bilayer, enabling integrated delivery of complex molecular signals to recipient cells. These properties raise the possibility that BEVs may contribute not only to spatial host–microbiome communication but also to the temporal regulation of host physiology. Although direct experimental evidence remains limited, this conceptual framework provides a compelling rationale for investigating BEVs as previously underappreciated regulators of circadian physiology and host–microbiome communication.

### Microbiome-Derived Signals Regulating Host Circadian Rhythms

The gut microbiota regulates host circadian physiology through a diverse repertoire of microbiome-derived signaling molecules that integrate microbial metabolism with host biological rhythms. These microbial products function as peripheral zeitgebers capable of synchronizing intestinal and systemic circadian clocks independently of the central light-entrained oscillator. Temporal fluctuations in microbial metabolism have emerged as important determinants of host metabolic homeostasis, immune regulation, and epithelial function (Fig. 3A) [7, 13, 97]. Among the currently identified microbiome-derived signals, SCFAs represent the best-characterized mediators of microbiome–circadian communication. Produced through bacterial fermentation of dietary fiber, acetate, propionate, and butyrate exhibit robust diurnal oscillations that closely follow feeding rhythms and microbial activity [98]. SCFAs regulate peripheral clock function through activation of G protein-coupled receptors, including GPR41 and GPR43, while also functioning as histone deacetylase (HDAC) inhibitors that influence chromatin accessibility and circadian gene expression [99, 100]. Through these mechanisms, SCFAs modulate the rhythmic expression of *BMAL1*, PER, and REV-ERBα, thereby influencing intestinal epithelial renewal, glucose metabolism, lipid utilization, and immune homeostasis.

Bile acids constitute another major class of microbiome-derived circadian regulators. Primary bile acids synthesized in the liver undergo extensive microbial biotransformation within the intestine to generate secondary bile acids, which activate nuclear receptors such as farnesoid X receptor (FXR) and Takeda G protein-coupled receptor 5 (TGR5) [101]. Because both bile acid synthesis and microbial metabolism exhibit pronounced circadian oscillations, bile acid signaling contributes to synchronizing hepatic and intestinal clocks with nutrient availability [102]. Dysregulation of bile acid rhythmicity has been associated with impaired glucose metabolism, hepatic steatosis, and chronic inflammation, emphasizing the importance of microbiota-dependent bile acid metabolism in circadian regulation.

The tryptophan metabolic pathway provides another important molecular link between the gut microbiota and host circadian physiology. Intestinal microorganisms convert dietary tryptophan into a diverse array of bioactive metabolites, including indole derivatives, indole-3-propionic acid, indole-3-acetic acid, and serotonin precursors [103]. These metabolites regulate intestinal immunity, epithelial barrier integrity, and neuroendocrine signaling through activation of the aryl hydrocarbon receptor (AhR) and other host signaling pathways [104]. In addition, serotonin synthesized within the gastrointestinal tract exhibits circadian rhythmicity and contributes to the regulation of gastrointestinal motility, feeding behavior, and peripheral metabolic function, further supporting the intimate relationship between microbial metabolism and host biological timing [105].

Beyond these major metabolites, numerous additional microbiome-derived factors, including polyamines, vitamins, amino acid metabolites, and bacterial signaling molecules, have also been implicated in circadian regulation [106]. These microbial metabolites establish an interconnected biochemical network that synchronizes microbial metabolism with host physiological rhythms [106]. Importantly, many of these signaling molecules exhibit time-dependent oscillations that parallel rhythmic changes in microbial abundance and metabolic activity, reinforcing the concept that temporal microbial metabolism contributes directly to circadian homeostasis [107].

Despite these important advances, current understanding of microbiome–circadian communication has largely focused on individual soluble metabolites acting through specific host receptors [108]. Although these studies have substantially advanced the field, they do not fully explain how the diverse array of microbial signals is coordinated, integrated, and delivered simultaneously to host tissues. Under physiological conditions, host cells are exposed to complex combinations of microbial proteins, nucleic acids, lipids, and metabolites rather than isolated signaling molecules [109]. This observation indicates additional mechanisms capable of integrating multiple microbial signals are likely to participate in temporal host–microbiome communication.

In this context, BEVs represent a fundamentally distinct mode of microbiome-derived signaling [2]. Rather than delivering individual metabolites, BEVs package proteins, lipids, nucleic acids, and metabolites within a single membrane-bound structure, enabling the coordinated transfer of multiple bioactive cargoes to epithelial cells, immune cells, and distant metabolic organs (Fig. 3B) [110]. This integrated mode of communication provides a conceptual framework for understanding how rhythmic microbial activity may be translated into coordinated host circadian responses. Although direct experimental evidence remains limited, BEVs offer a compelling mechanistic hypothesis that extends beyond conventional metabolite-centered models and establishes a new perspective on how temporal microbial information may be transmitted throughout the gut microbiome–circadian axis.

### BEVs as Emerging Temporal Signaling Mediators

Although microbiome-derived metabolites have long been regarded as the principal mediators of microbiome–circadian communication, they are unlikely to fully explain how temporally coordinated microbial information is integrated and transmitted to host tissues [106]. Within the intestinal environment, host cells are continuously exposed to a complex repertoire of microbial molecules including proteins, lipids, nucleic acids, and metabolites that collectively regulate immune responses, epithelial homeostasis, and metabolic function. Understanding how these diverse microbial signals are coordinated and delivered to host tissues has therefore become an important challenge in microbiome research. In this context, BEVs represent a fundamentally distinct class of microbiome-derived signaling mediators that differ from conventional soluble microbial products [25]. Unlike individual metabolites that generally activate specific receptors or signaling pathways, BEVs encapsulate multiple classes of bioactive cargo within a single lipid bilayer-enclosed structure, enabling the coordinated delivery of proteins, lipids, nucleic acids, and metabolites to recipient cells. This integrated mode of cargo delivery allows multiple biological pathways to be regulated simultaneously, supporting the concept that BEVs function as coordinated signaling platforms rather than simple extracellular transport vehicles.

The membrane-bound architecture of BEVs provides several biological advantages that may facilitate host–microbiome communication and could potentially support temporal signaling. Encapsulation protects vesicular cargo from enzymatic degradation within the gastrointestinal tract and circulation while preserving the biological activity of proteins, nucleic acids, and metabolites during transport [16]. Furthermore, BEVs readily interact with intestinal epithelial cells, macrophages, dendritic cells, and other immune populations through membrane fusion, endocytosis, and receptor-mediated uptake, enabling efficient intracellular delivery of multiple bioactive molecules. Unlike freely diffusible metabolites, which generally regulate individual signaling pathways independently, BEVs deliver coordinated molecular signals capable of simultaneously influencing epithelial barrier integrity, immune responses, microbial ecology, and metabolic pathways.

An additional characteristic distinguishing BEVs from conventional microbiome-derived signaling molecules is their capacity for selective cargo loading. Proteomic, lipidomic, and transcriptomic studies have demonstrated that vesicular cargo is not randomly incorporated during vesicle formation but is selectively enriched according to bacterial species, environmental conditions, and physiological status [41]. Consequently, the molecular composition of BEVs reflects the metabolic activity of the parental bacterium and dynamically adapts to environmental stimuli. Because bacterial metabolism, gene expression, and microbial community activity exhibit pronounced diurnal oscillations, it is plausible that BEV composition and biological activity may also undergo temporal regulation. However, direct experimental evidence demonstrating time-of-day-dependent changes in BEV biogenesis or cargo loading remains limited. Thus, the potential temporal regulation of BEVs should be considered a testable hypothesis rather than an established mechanism.

Recent studies have revealed a striking convergence between biological processes regulated by BEVs and those governed by the circadian clock. BEVs regulate intestinal epithelial renewal by modulating tight junction proteins and mucus production, influence innate and adaptive immune responses through Toll-like receptor (TLR) and NF-κB signaling, shape microbial community composition, and regulate glucose and lipid metabolism in peripheral tissues [2, 23, 50, 51]. Notably, these physiological processes are themselves under strong circadian regulation and display robust daily oscillations. Rather than functioning independently of circadian biology, BEV-mediated signaling may intersect with endogenous clock-controlled pathways and thereby contribute to the temporal regulation of host physiology. Although direct evidence linking BEVs to circadian regulation remains limited, probiotic-derived BEVs have been shown to improve epithelial barrier integrity, suppress inflammatory cytokine production, and modulate host metabolic responses through signaling pathways closely associated with circadian physiology, including NF-κB, AMPK, and PPAR signaling [111, 112]. Given the central roles of these pathways in coordinating immune, metabolic, and circadian homeostasis, these findings support the hypothesis that BEVs may indirectly influence host circadian regulation through orchestrated modulation of epithelial function, immune responses, and gut microbial ecology. However, direct evidence demonstrates that BEVs regulate core molecular clock components, such as *BMAL1*, *CLOCK*, or PER2, or exhibit time-of-day-dependent changes in vesicle abundance, biogenesis, or cargo loading remains lacking. These unresolved questions highlight an important direction for future investigation.

Thus, current evidence provides a rationale for a conceptual model in which BEVs may serve as emerging temporal signaling mediators within the gut microbiome–circadian axis (Fig. 3B). Rather than acting as isolated signaling molecules, BEVs integrate proteins, lipids, nucleic acids, and metabolites into a coordinated extracellular platform capable of synchronizing microbial ecology with epithelial barrier integrity, immune regulation, and metabolic homeostasis. This integrated mode of communication provides a biologically plausible mechanism by which rhythmic microbial activity could potentially be translated into coordinated host physiological responses. However, whether BEV abundance, biogenesis, cargo composition, or host responsiveness exhibits circadian or time-of-day-dependent regulation remains to be experimentally determined. Determining whether vesicle biogenesis, cargo composition, and host responsiveness are themselves subject to circadian regulation now represents an important frontier in BEV biology and may provide new therapeutic opportunities for inflammatory and metabolic disease (Fig. 3).

### Potential Roles of BEVs in Circadian-Associated Diseases

Disruption of the gut microbiome–circadian axis is increasingly recognized as a critical contributor to the development of chronic inflammatory and metabolic diseases. Circadian misalignment alters microbial composition, epithelial barrier integrity, immune regulation, and metabolic homeostasis, thereby increasing susceptibility to a broad spectrum of disorders characterized by persistent inflammation and metabolic dysfunction. Because BEVs regulate many of these circadian-controlled biological processes, they represent potential candidates for linking microbiome-derived signals with circadian-associated disease processes. Although direct evidence connecting BEVs to circadian regulation remains limited, accumulating studies suggest that vesicle-mediated signaling converges on key physiological pathways governed by the host circadian system.

Among circadian-associated disorders, IBD represents one of the best-characterized examples of disrupted temporal host–microbiome interactions [113]. Clinical and experimental studies have demonstrated that patients with IBD exhibit altered expression of core clock genes together with impaired microbial rhythmicity and intestinal barrier dysfunction [114]. Circadian disruption further aggravates intestinal inflammation by enhancing epithelial permeability, promoting pro-inflammatory cytokine production, and altering immune cell recruitment [97]. Numerous studies have shown that probiotic-derived BEVs improve epithelial barrier integrity by upregulating tight junction proteins, including occludin, claudins, and zonula occludens-1 (ZO-1), while simultaneously suppressing inflammatory signaling pathways such as NF-κB [50, 51]. These observations suggest that BEVs may indirectly contribute to the restoration of circadian homeostasis through balanced regulation of epithelial function and immune responses. Although no study has yet demonstrated that probiotic-derived BEVs directly regulate molecular clock genes in experimental colitis, accumulating evidence indicates that BEVs target biological processes that are themselves tightly controlled by circadian rhythms, including epithelial barrier maintenance, innate immune responses, inflammatory signaling, and host metabolism [115]. Modulation of these circadian-associated pathways provides a plausible mechanistic basis through which BEVs may contribute to the restoration of temporal homeostasis during intestinal inflammation. Future studies investigating the direct effects of BEVs on molecular clock networks will be essential to establish their role in circadian regulation.

The gut–skin axis has also emerged as an important component of circadian biology [116]. Skin physiology exhibits pronounced circadian oscillations that regulate epidermal differentiation, barrier function, oxidative stress responses, and immune surveillance [117]. Disturbance of these rhythms has been implicated in inflammatory skin disorders, including atopic dermatitis (AD) [118]. Recent evidence indicates that gut microbial dysbiosis contributes to AD pathogenesis through alterations in microbial metabolites, immune signaling, and neuroendocrine pathways [119]. Probiotic-derived BEVs have been shown to alleviate experimental AD by modulating gut microbial composition, reducing inflammatory cytokine production, and regulating serotonin-associated pathways within the intestine and skin [47]. Although direct evidence linking these effects to circadian regulation remains limited, serotonin metabolism and skin barrier function are themselves under circadian control, suggesting that BEV-mediated modulation of the gut–skin axis may influence disease progression through physiological pathways that are themselves subject to circadian regulation [120, 121].

Increasing attention has likewise been directed toward the role of circadian disruption in metabolic dysfunction-associated steatotic liver disease (MASLD) and other metabolic disorders. Hepatic lipid metabolism, glucose homeostasis, and inflammatory responses are tightly regulated by the circadian clock, and disruption of hepatic clock function accelerates steatosis and metabolic inflammation [11, 122]. Gut microbiota-derived signals contribute substantially to gut–liver communication, with dysbiosis promoting increased intestinal permeability and translocation of microbial products into the portal circulation [123, 124]. BEVs released by beneficial bacteria have recently been reported to ameliorate experimental MASLD by modulating gut microbial composition, restoring intestinal barrier integrity, and regulating hepatic lipid metabolism [125]. These improvements are accompanied by reduced hepatic inflammation and enhanced lipid oxidation, biological processes that are themselves under circadian regulation. Although direct evidence linking BEVs to hepatic circadian signaling is currently lacking, these observations support the hypothesis that BEVs may contribute to metabolic homeostasis through modulation of microbiome-derived signals along the gut–liver axis.

Collectively, these disease models indicate that BEVs consistently target biological processes governed by the host circadian system rather than isolated pathological events. Across intestinal, cutaneous, and hepatic disorders, BEVs regulate epithelial barrier integrity, immune homeostasis, microbial ecology, and metabolic function, all of which are tightly integrated with circadian physiology. However, direct evidence demonstrating rhythmic BEV biogenesis, time-dependent cargo dynamics, or circadian-dependent vesicle signaling remains limited. Taken together, the convergence of these findings provides a rationale for considering BEVs as potential temporal signaling mediators within the gut microbiome–circadian axis, although this proposed role remains to be experimentally validated. A deeper understanding of reciprocal interactions between BEVs and host circadian networks may ultimately facilitate the development of chronobiology-based microbiome therapeutics for inflammatory and metabolic diseases.

## Conclusion and Future Perspective

BEVs are increasingly recognized as key mediators of host–microbiome communication, facilitating interkingdom signaling through the coordinated delivery of diverse bioactive cargo. Encapsulation within a stable lipid bilayer protects proteins, lipids, nucleic acids, and metabolites from extracellular degradation while enabling their simultaneous delivery to recipient cells, thereby influencing multiple physiological processes. Unlike conventional microbiome-derived metabolites that generally regulate individual signaling pathways, BEVs integrate multiple classes of molecular signals into a single biological platform capable of coordinating epithelial barrier function, immune homeostasis, microbial ecology, and metabolic regulation. This unique biological organization positions BEVs as an emerging signaling system within the gut microbiome–host communication network.

This review highlights the emerging concept that vesicle-mediated signaling may provide a previously underappreciated connection between the gut microbiome and the host circadian system. Microbial metabolites, including short-chain fatty acids, bile acids, and tryptophan derivatives, are well-established regulators of circadian physiology. In contrast, vesicle-mediated signaling offers a complementary mechanism by integrating multiple classes of bioactive molecules into a single delivery system. This integrated mode of cargo delivery raises the possibility that microbial vesicles contribute not only to spatial communication between the gut microbiota and the host but also to the temporal signaling within the gut microbiome-circadian axis. Although probiotic- and commensal-derived BEVs have shown therapeutic potential in multiple inflammatory and metabolic disease models, their direct role in circadian regulation remains largely unexplored. Most studies have evaluated BEV function under static experimental conditions. Consequently, little is known about the temporal regulation of vesicle biogenesis, cargo selection, tissue distribution, or host responsiveness. Whether BEV abundance or cargo composition varies according to time of day, or whether BEVs directly influence core clock components such as *BMAL1*, *CLOCK*, and PER2, remains to be determined. Moreover, methodological variability across bacterial strains, culture conditions, isolation procedures, and characterization strategies continues to limit reproducibility and cross-study comparisons. Establishing standardized experimental workflows and reporting guidelines will therefore be critical for advancing mechanistic understanding and accelerating the clinical translation of BEV research.

Future studies should combine multi-omics approaches with circadian biology, spatial transcriptomics, and temporal sampling strategies to define how BEV production, cargo composition, and biological activity change over time. Particular attention should be directed toward determining whether vesicle release and cargo loading exhibit circadian oscillations, identifying cargo molecules responsible for clock regulation, and elucidating the mechanisms through which vesicular signals interact with host circadian pathways. Addressing these questions will deepen our understanding of temporal host–microbiome communication and provide mechanistic insight into inflammatory and metabolic diseases. From a translational perspective, BEVs represent promising next-generation postbiotic platforms because of their intrinsic stability, selective cargo loading, and natural capacity for interkingdom communication. Beyond serving as passive carriers of bacterial molecules, BEVs should be viewed as integrated signaling platforms capable of coordinating both spatial and temporal communication between the gut microbiota and the host. As knowledge of the gut microbiome–circadian axis continues to expand, these unique biological properties may be harnessed to develop chronobiology-guided microbiome interventions aimed at restoring immune, metabolic, and circadian homeostasis.

In conclusion, this review proposes a conceptual framework in which BEVs may serve as potential temporal signaling mediators linking the gut microbiome with the host circadian system. By integrating diverse microbial signals into coordinated extracellular communication networks, BEVs extend the current paradigm of microbiome–host signaling beyond conventional metabolite-centered mechanisms. However, direct evidence demonstrating diurnal changes in BEV abundance, biogenesis, cargo dynamics, or direct regulation of core clock components remains limited. Therefore, the proposed role of BEVs as temporal signaling mediators should be considered a testable hypothesis that requires further experimental validation. Elucidating whether vesicle biogenesis, cargo dynamics, and host responsiveness are themselves subject to circadian regulation represents an important frontier in microbiome research and may open new opportunities for the development of chronobiology-guided diagnostics and therapeutics for inflammatory and metabolic diseases.

## Figures and Tables

**Table 1:** Comparison of representative methods for the isolation and purification of bacterial extracellular vesicles.

Isolation method	Principle	Yield	Purity	Vesicle Integrity	Typical applications	Ref.
Differential centrifugation	Sedimentation by centrifugal force	High	Moderate	Good	General isolation	[126, 127]
Density gradient ultracentrifugation	Separation by buoyant density	Moderate	Very high	Excellent	Proteomics Lipidomics	[128, 129]
Ultrafiltration	Membrane filtration	High	Moderate	Moderate	Rapid concentration	[78, 130]
Size exclusion chromatography	Size-based separation	Moderate	High	Excellent	Functional studies	[131, 132]
PEG precipitation	Polymer-mediated precipitation	Very high	Moderate	Good	Large-scale isolation	[133, 134]

**Table 2:** Representative evidence for the roles of bacterial extracellular vesicles in host–microbiome communication.

EV sourced bacteria	Disease	Description	Ref.
*Clostridium butyricum*	Ulcerative colitis	- Suppresses colitis via macrophage polarization (M1 → M2) - Reshapes gut microbiota composition - Reduces intestinal inflammation	[135]
*Roseburia intestinalis*	Colitis	- Enhances intestinal barrier integrity (tight junctions) - Modulates gut microbiota composition - Decreases inflammatory cytokines	[78]
*Lactobacillus rhamnosus* GG	Colorectal cancer	- Enhances anti-PD-1 immunotherapy efficacy - Improves tumor immune microenvironment - Promotes anti-tumor immune response - Modulates macrophage phenotype (anti-inflammatory shift)	[82]
*Limosilactobacillus mucosae*	Diarrhea	- Maintains gut homeostasis - Reduces intestinal inflammation	[77]
*Lactobacillus gasseri*, *Gardnerella vaginalis*	Vaginal infection (*Trichomonas vaginalis*)	- Regulates vaginal microbiome composition - Influences host-pathogen interactions - Modulates infection susceptibility	[79]
Bacteroides thetaiotaomicron	IBD	- Alters responses under IBD conditions - Impacts intestinal immune homeostasis	[74]
